# Electroencephalographic Response to Sodium Nitrite May Predict Delayed Cerebral Ischemia After Severe Subarachnoid Hemorrhage

**DOI:** 10.1097/CCM.0000000000001950

**Published:** 2016-10-14

**Authors:** Payashi S. Garry, Matthew J. Rowland, Martyn Ezra, Mari Herigstad, Anja Hayen, Jamie W. Sleigh, Jon Westbrook, Catherine E. Warnaby, Kyle T. S. Pattinson

**Affiliations:** 1 Nuffield Department of Clinical Neurosciences, University of Oxford, Oxford, United Kingdom.; 2 Neurosciences Intensive Care Unit, Neurosciences, Orthopaedics, Trauma and Specialist Surgery, Oxford University Hospitals NHS Trust, John Radcliffe Hospital, Oxford, United Kingdom.; 3 Department of Clinical Health Care, Oxford Brookes University, Oxford, United Kingdom.; 4 School of Psychology and Clinical Language Sciences, University of Reading, Reading, United Kingdom.; 5 Department of Anaesthesia, University of Auckland, Waikato Hospital, Hamilton, New Zealand

**Keywords:** aneurysm, intracranial berry, brain injuries, electroencephalography, nitric oxide donors, spectrum analysis, subarachnoid hemorrhage

## Abstract

**Objectives:**

Aneurysmal subarachnoid hemorrhage often leads to death and poor clinical outcome. Injury occurring during the first 72 hours is termed “early brain injury,” with disruption of the nitric oxide pathway playing an important pathophysiologic role in its development. Quantitative electroencephalographic variables, such as α/δ frequency ratio, are surrogate markers of cerebral ischemia. This study assessed the quantitative electroencephalographic response to a cerebral nitric oxide donor (intravenous sodium nitrite) to explore whether this correlates with the eventual development of delayed cerebral ischemia.

**Design:**

Unblinded pilot study testing response to drug intervention.

**Setting:**

Neuroscience ICU, John Radcliffe Hospital, Oxford, United Kingdom.

**Patients:**

Fourteen World Federation of Neurosurgeons grades 3, 4, and 5 patients (mean age, 52.8 yr [range, 41–69 yr]; 11 women).

**Interventions:**

IV sodium nitrite (10 μg/kg/min) for 1 hour.

**Measurements and Main Results:**

Continuous electroencephalographic recording for 2 hours. The alpha/delta frequency ratio was measured before and during IV sodium nitrite infusion. Seven of 14 patients developed delayed cerebral ischemia. There was a +30% to +118% (range) increase in the alpha/delta frequency ratio in patients who did not develop delayed cerebral ischemia (*p* < 0.0001) but an overall decrease in the alpha/delta frequency ratio in those patients who did develop delayed cerebral ischemia (range, +11% to –31%) (*p* = 0.006, multivariate analysis accounting for major confounds).

**Conclusions:**

Administration of sodium nitrite after severe subarachnoid hemorrhage differentially influences quantitative electroencephalographic variables depending on the patient’s susceptibility to development of delayed cerebral ischemia. With further validation in a larger sample size, this response may be developed as a tool for risk stratification after aneurysmal subarachnoid hemorrhage.

## Abstract 2

Supplemental Digital Content is available in the text.

Subarachnoid hemorrhage (SAH) is usually caused by rupture of a cerebral aneurysm located in the circle of Willis (1). It disproportionately affects a younger population (2) and is often fatal. The main mechanisms of brain injury after SAH include early brain injury (EBI), which occurs in the first 72 hours following ictus, and delayed cerebral ischemia (DCI), in 30% of patients unpredictably 3–14 days later (3). It is difficult to predict the evolution of cerebral injury following SAH using currently available methods. Clinical scoring systems have poor accuracy, and bedside clinical monitoring requires specialist interpretation.

The focus of SAH research has been to investigate the mechanisms behind DCI, as this remains the most important cause of morbidity and mortality in patients who survive initial aneurysm rupture (4). Historically, DCI was attributed to spasm of the cerebral blood vessels; however, the relationship between angiographic evidence of vessel spasm and DCI is weak (5, 6). In addition, treatment of cerebral arterial vasoconstriction does not improve clinical outcome (7). There is now a growing body of evidence suggesting that changes occurring during EBI set the scene for the development of DCI (3, 7). An improved understanding and measurement of the processes associated with EBI could offer an opportunity to better predict DCI and improve patient outcomes (8).

Emerging evidence suggests that disruption to the nitric oxide (NO) signaling pathway may play a critical role in EBI (9–11). Endogenous NO has been proposed to exert a protective action after brain injury through a number of different pathways, including promoting cerebral blood flow, attenuating mitochondrial damage, and preventing cellular apoptosis (12). Following SAH, the integrity of these pathways becomes disrupted, resulting in a cascade of cellular injury resulting in cell death (13). The level of disruption of the NO signaling pathways has been shown to correlate with eventual outcome after SAH (10).

Sodium nitrite (a prodrug) is suited as an exogenous NO donor in SAH patients because it is converted to NO under conditions of hypoxia or acidosis. As sodium nitrite has a relatively rapid onset of action, its effects are measurable in a brief time frame and, when combined with a measurement of cerebral injury, it may represent an ideal drug probe of EBI severity.

Quantitative electroencephalography (qEEG) uses power spectral analysis to obtain measures of the different components of the electroencephalography, sensitively detecting disturbed neuronal activity during the development of ischemia (14). SAH results in a variety of abnormalities in qEEG variables, specifically decreases in the alpha/delta frequency ratio (ADR), and a decrease in relative alpha power variability (15–18). These patterns have been shown to correlate with subsequent cerebral ischemia (19).

Based on the evidence that disruption of the cerebral NO pathway is a main driver of the pathophysiologic processes occurring during EBI, we hypothesized that the qEEG response to a sodium nitrite, an NO donor, could be used as a dynamic probe of EBI severity and that this response would be linked to the subsequent development of DCI.

## MATERIALS AND METHODS

Patients 18–80 years old admitted to the neuroscience ICU (NICU) at the John Radcliffe Hospital, Oxford, after having suffered severe aneurysmal SAH (World Federation of Neurosurgeons [WFNS] grade 3, 4, or 5 at the time of presentation) were eligible for inclusion in the study. No patient showed clinical evidence of DCI or angiographic cerebral arterial constriction at the time of the study.

Written informed consent was obtained from the next of kin of all participants and from participants if they regained capacity to consent. The study was approved by the South Central—Oxford C NHS Health Research Authority Ethics Committee 12/SC/0366. Exclusion criteria included contraindications to sodium nitrite, specifically severe cardiovascular compromise and preexisting methemoglobinemia. Next of kin provided information regarding smoking, medication, hypertension, and family history.

All patients underwent standard clinical care that was not influenced by inclusion in this study. All were given nimodipine for 14 days. Computed tomography (CT) of the brain was performed in the event of lack of wakening in sedated patients or worsening of neurologic signs in awake patients, according to the clinical policy of the neuro-ICU. Patients who did not demonstrate neurologic deterioration did not undergo CT scanning in the acute period; however, they did later receive follow-up brain magnetic resonance imaging at 6 months as per local protocol, which confirmed the lack of new infarction.

DCI was diagnosed based on consensus guidelines (20). In those patients who remained intubated and sedated, this was done by CT. Two patients (patients 2 and 4) had treatment withdrawn and subsequently died after CT evidence of widespread infarction secondary to DCI. The study investigators were not responsible for the clinical care of these patients. Treatment of DCI was via a standardized protocol involving hypertension, maintenance of euvolemia, and maintenance of a hemoglobin level above 8 g/dL.

### Study Design

#### Electroencephalography.

Following definitive endovascular aneurysm treatment, each patient underwent a 2-hour period of continuous electroencephalographic monitoring (Porti 7 system, Twente Medical Systems International, Oldenzaal, The Netherlands) on one occasion, as soon as possible after endovascular securing of the aneurysm. We used a simplified electroencephalographic montage (16), a compromise between stable maintenance of recording and full coverage of all vascular territories. Seven to 13 unipolar electroencephalographic electrodes were used at the following positions defined according to the international 10–20 system: Cz, Fz, Pz, Fp1, Fp2, F3, F4, P3, P4, T3, T4, O1, and O2. Electroencephalographic data were digitized at a sampling rate of 2,048 Hz, with a high pass filter of 0.5 Hz and a low pass filter of 30 Hz.

#### Sodium Nitrite Infusion.

An infusion of sodium nitrite at 10 μg/kg/min was commenced at the start of the second hour of electroencephalographic recording and continued for 1 hour. The dosing schedule was developed as a compromise between ensuring adequate delivery of cerebral NO and minimization of cardiovascular effects. Changes in infusion rates of sedative drug levels and vasopressors were minimized for the duration of the recording.

#### Physiologic Measurements.

Participants underwent simultaneous transcranial Doppler (TCD) monitoring. Insonation of the middle cerebral artery (MCA) M1 segment was performed unilaterally on the side with the best window using color-coded duplex ultrasound (EZ-Dop, DWL; 2-Mz probe; EZ-Dop GmbH, Singen, Germany). End-tidal Co_2_, end-tidal O_2_, arterial blood pressure, and pulse oximetry were recorded continuously and collected on a Power-1401 data acquisition interface (Cambridge Electronic Design, Cambridge, UK). Arterial Co_2_ values were collected once during the duration of the recording as part of routine clinical care. Missing values are as follows: patient 10 did not have an adequate TCD window and we were unable to record the intracranial pressure (ICP) waveform for patients 1 and 13 and end-tidal gases in patient 7.

#### Follow-Up.

Each surviving patient was followed up at 3–6 months post rupture. Telephone follow-up was performed for patients who were unable to attend hospital. Primary outcome was defined as the presence or absence of DCI. Secondary outcome was assessed by modified Rankin scale (21) at 3 months, via structured standardized questions in person or by telephone.

### Quantitative Electroencephalographic Analysis

Preprocessing was carried out using custom-written MATLAB (MathWorks, Natick, MA) code and the EEGLAB version 13.4.3b analysis toolbox (EEGLAB, San Diego, CA) (22). Datasets were rereferenced to the average of mastoid reference electrodes and band pass filtered from 0.5 to 15 Hz using a linear finite impulse response filter. Each electroencephalographic recording was visually inspected, and artifacts were manually removed.

Spectral analysis was carried out using FieldTrip (23), a MATLAB software toolbox for electroencephalographic analysis. Data were windowed into 30-second segments that overlapped by 50%. Time-frequency analysis was performed using a multitaper spectral estimation using discrete prolate spheroidal (Slepian) sequences with 14 tapers and fast Fourier transform algorithm for each electrode channel. Five 60-second epochs were selected randomly from the first (baseline) and last 30 minutes of the recording (during infusion). The epochs were separated by at least 60 seconds to avoid autocorrelation. The corresponding frequency distribution in each epoch was identified, which enabled determination of power values in the following frequency bands: delta, 1–4 Hz; alpha, 8–12 Hz; and total low frequency power, 1–15 Hz.

Two multivariate analyses were used to investigate the effects of sodium nitrite on ADR, including potential confounding factors as covariates (age, propofol, midazolam, and WFNS grade, using the R statistical package; R Foundation for Statistical Computing, Vienna, Austria). The square root of the ADR (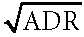) was used as the response variable to achieve the required normality and homoscedasticity in the residuals. The goodness of fit was assessed via Shapiro-Wilk normality tests on fixed and random effect residuals and by calculation of correlation coefficient (*R*^2^).

A multilevel linear regression model incorporated both baseline ADR and the ADR response to sodium nitrite as response variables. This model takes into account the repeated measures taken on each patient. A second model incorporated baseline 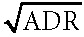 as a covariate, with the ADR response to sodium nitrite as a response. This model used a Bayesian approach, allowing the baseline 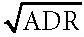 to be incorporated as a normally distributed random variable. This enabled direct investigation of the effect of sodium nitrite on the baseline 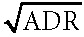 and allowed us to explore whether the dependence of the sodium nitrite effect on baseline 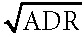 for patients who did not develop DCI differed significantly from those that subsequently developed DCI. Further description of these approaches can be found in the **supplementary information** (Supplemental Digital Content 1, http://links.lww.com/CCM/B945).

### Physiologic Data Analysis

Waveform analysis was performed using custom-written MATLAB code, enabling calculation of average baseline and nitrite infusion values for TCD MCA velocity (MCAV), arterial blood pressure, end-tidal Co_2_, end-tidal O_2_, and ICP. Values were compared for each subject using paired *t* tests. *p* values less than 0.05 were considered significant.

### Sample Size Calculation

As a study such as this has not been performed before, a formal power calculation was not possible. We anticipated a powerful effect on electroencephalographic power based on a previous resting electroencephalographic study, which showed a 24% lower ADR in nine patients who developed DCI (15). Animal studies have demonstrated that sodium nitrite seems to have a strong effect on the subsequent development of ischemia (24). We therefore expected 10–14 patients to demonstrate enough change to allow an effect of the drug to be detected.

## RESULTS

### Demographics, Treatment and Clinical Outcomes

Fourteen patients (mean age, 52.8 yr [range, 41–69 yr]; 11 women) with spontaneous SAH successfully treated with endovascular coiling were recruited over a total study period of 13 months. All patients admitted to the NICU at the John Radcliffe Hospital were eligible for inclusion in the study.

All patients had modified Fisher grade 4 (thick SAH with intraventricular hemorrhage) and WFNS grades 3–5 on initial presentation. The WFNS grade 3 patients (patients 5, 7, 9, 10, and 12) were sedated and intubated either because of a subsequent drop in their Glasgow Coma Score secondary to seizures or subsequent episodes of vomiting. Detailed information on patient demographics and complications and levels of sedative drugs and vasopressors can be found in the supplementary information (Supplemental Digital Content 1, http://links.lww.com/CCM/B945).

Data were collected between 2 and 4 days (mean, 3.5 d) following primary SAH. Because of cardiovascular instability and unknown behavior of sodium nitrite in this population at that time, it was not possible to collect data sooner than day 4 in patients 2 and 6. Seven of the study patients (50%) developed DCI as defined by consensus guidelines (20). This is in keeping with a higher incidence of DCI reported in previous studies of high-grade (WFNS grade, 3–5) SAH patients (25) (**Table 1**).

**TABLE 1.**
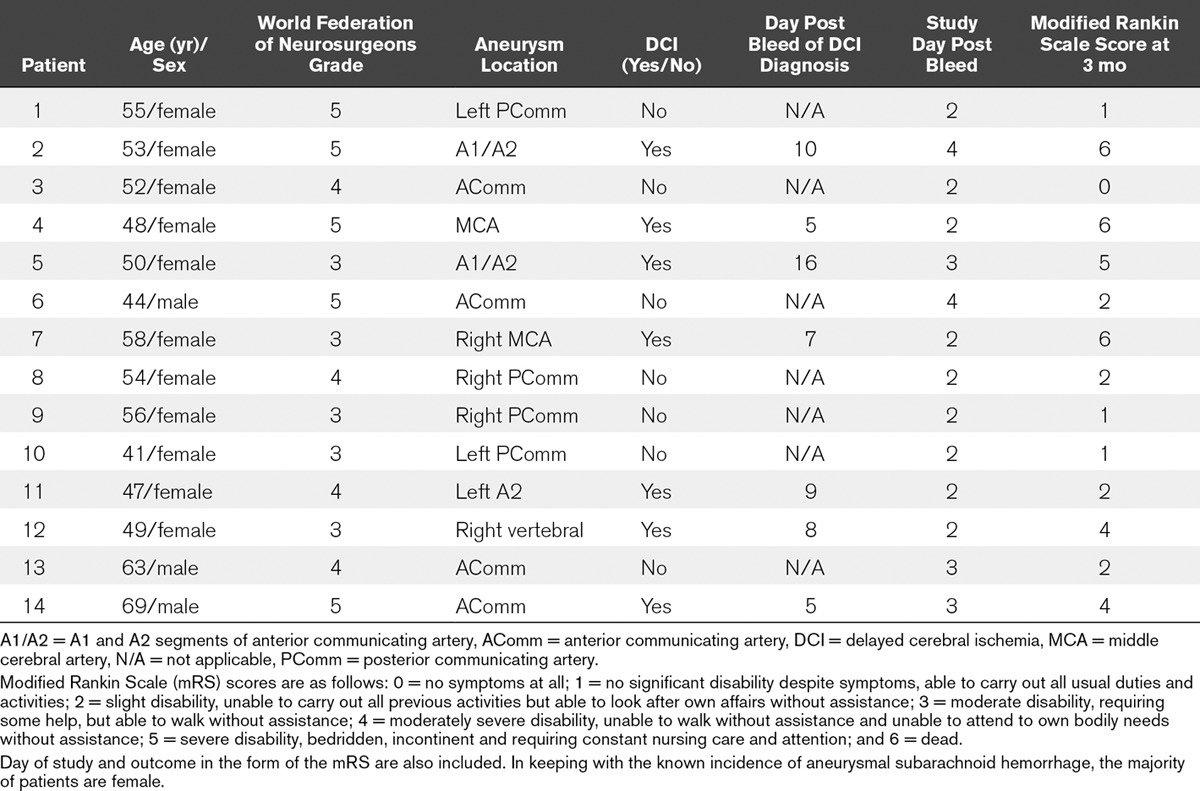
Demographics of Patients Recruited to the Study, Showing Age, World Federation of Neurosurgeons Grade, Aneurysm Location, and the Development of Delayed Cerebral Ischemia

All patients were diagnosed with hydrocephalus and were treated with external ventricular drainage immediately at admission to the neurosurgical centre. There was no rebleeding. All patients were treated with endovascular embolization. Three patients died (patients 2, 4, and 7), two from complications following severe DCI and one from cardiovascular instability. Three patients (patients 1, 5, and 14) developed sepsis secondary to chest infection, which were treated with IV antibiotics.

### qEEG Results

Visual inspection of raw electroencephalographic data did not reveal any ictal or preictal activity in any of the recruited patients.

**Figure 1.**
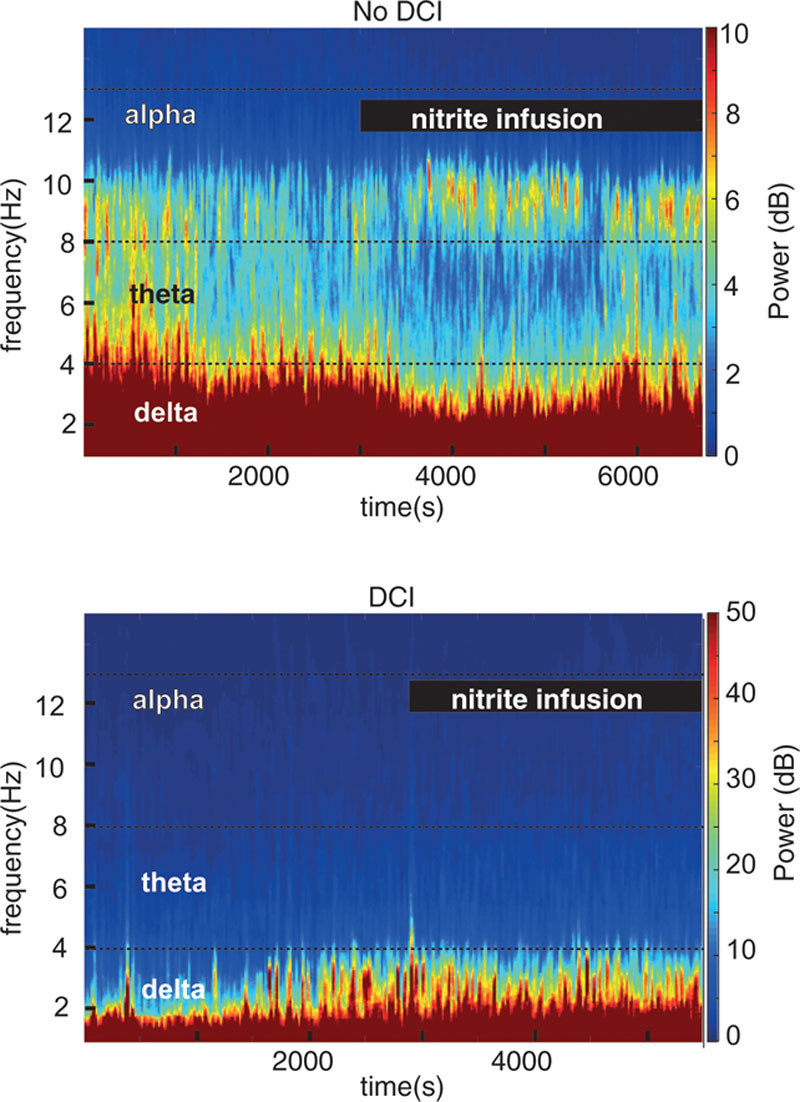
Single-channel spectrograms from a patient that did not develop delayed cerebral ischemia (DCI) (*top*) and from a patient who developed DCI (*bottom*).

**Figure 2.**
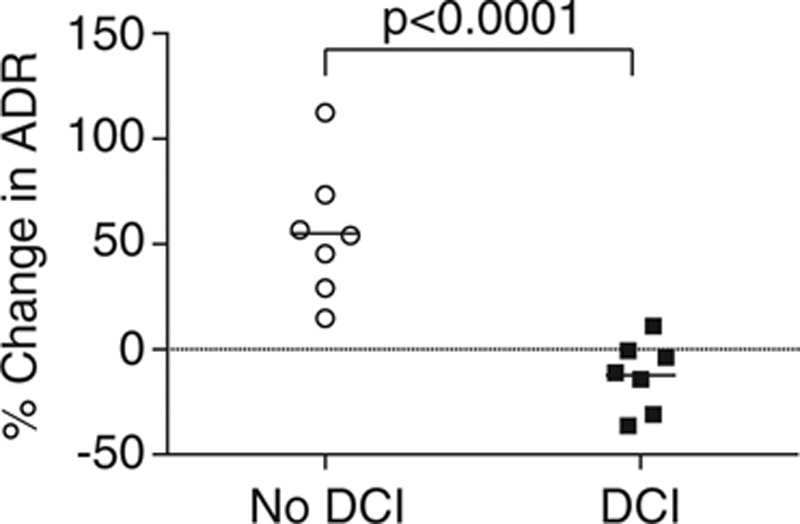
Scatter plot showing the percentage change in alpha/delta frequency ratio (ADR) for each patient, grouped by the presence or absence of subsequent delayed cerebral ischemia (DCI). The horizontal bar represents the mean for each group. It can be seen that there is greater variability in the response to the drug in the group that did not develop DCI, suggesting that there may be a spectrum of neuronal damage that is unmasked by the drug, raising the possibility that there may be differing degrees of neuronal disruption after severe subarachnoid hemorrhage.

**Figure 3.**
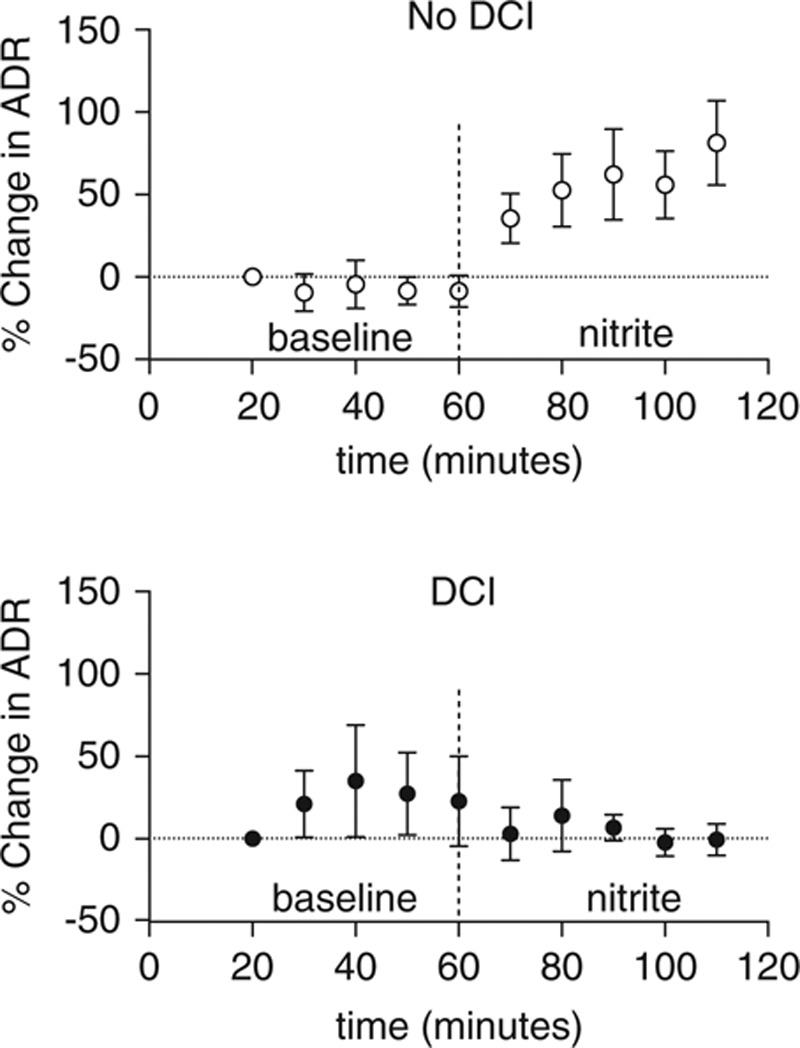
Percentage change from baseline alpha/delta frequency ratio (ADR) over time, calculated by dividing the ADR at each time point by the baseline for the patients who subsequently did not develop delayed cerebral ischemia (DCI) (*top*) and for those who did develop DCI (*bottom*). Error bars represent the sem. The dashed vertical line represents the start of the sodium nitrite infusion.

Spectrograms of the entire recording for two patients are shown in **Figure 1**. **Figure 2** illustrates the raw data from each patient represented as an average of the five values before and during infusion, which are converted into a percentage change from baseline. **Figure 3** demonstrates the percentage change from baseline ADR over time for the two groups.

Results from the linear regression model showed an increase in ADR from a mean of 0.033 (se = 0.008) to a mean of 0.055 (se = 0.010) in response to sodium nitrite in the no-DCI group (*p* < 0.0001) and a decrease in ADR from mean baseline of 0.056 (se = 0.010) to a mean of 0.050 (se = 0.009) in response to sodium nitrite in the DCI group (*p* = 0.006). There was a trend for a higher baseline ADR in the DCI group than the no-DCI group (*p* = 0.072). There was no evidence of an effect of propofol, midazolam, age, or WFNS grade on the ADR response. Comparing the change in ADR for the DCI group with the change in ADR in the no-DCI group demonstrated that the estimated mean for the DCI group was 0.028 (se = 0.003) less than that of the no-DCI group (*p <* 0.0001).

The Bayesian model confirmed the results described above and revealed that the baseline ADR had a significant effect on the ADR response to the drug. More specifically, it showed that in the no-DCI group, the baseline ADR increases by 0.273 
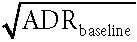
, whereas in the DCI group, the baseline ADR decreases by 
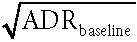
 in response to the drug. This model therefore further clarified that the ADR response to the drug was significantly affected by whether the patient subsequently developed DCI.

### Physiologic Data

In response to sodium nitrite infusion, there was a significant decrease (*p* = 0.026) in mean arterial pressure (MAP) from a mean of 87 mm Hg (sd, 13 mm Hg) to 84 mm Hg (sd, 13 mm Hg). There were no significant changes in MCAV, ICP, end-tidal Co_2_, or end-tidal O_2_ values (**Table 2**).

**TABLE 2.**
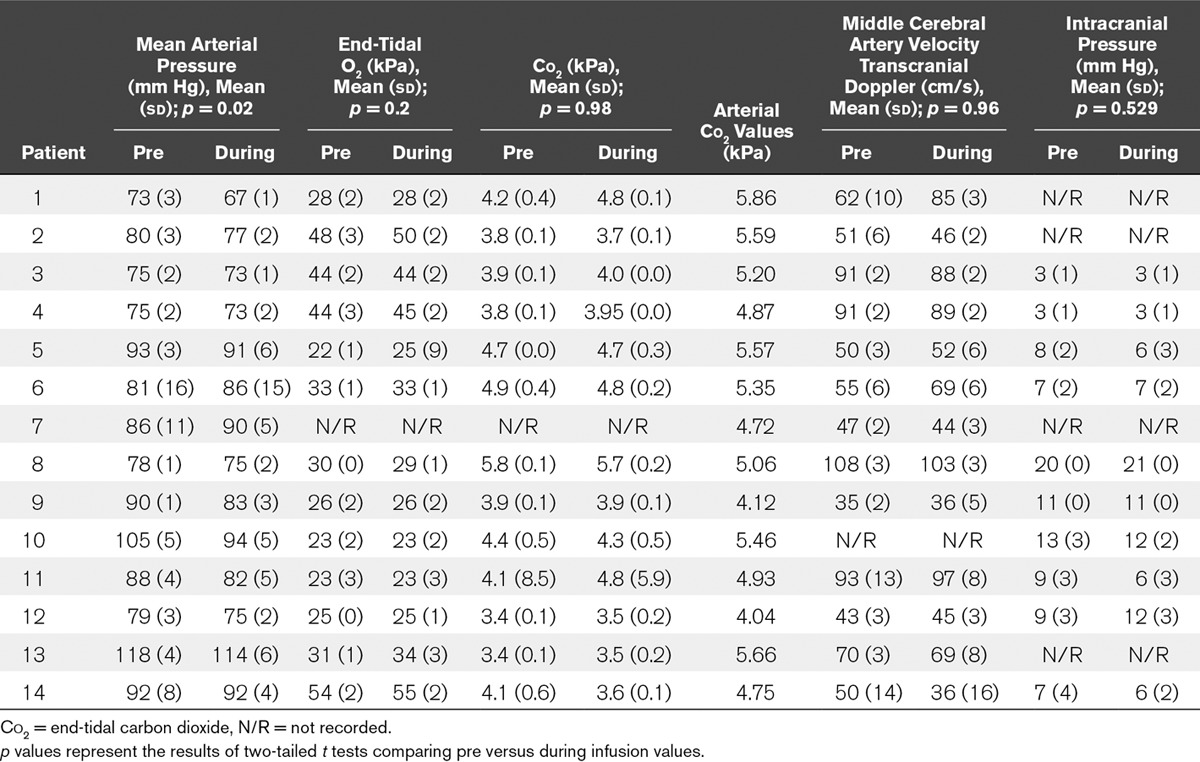
Mean Physiologic Values Pre Versus During Sodium Nitrite Infusion

## DISCUSSION

This pilot study investigated the qEEG response to IV sodium nitrite as a potential way to predict the development of DCI in patients with severe SAH. Patients who did not develop DCI showed a strong increase in the ADR in response to sodium nitrite, whereas patients who went on to develop DCI showed a small decrease or no change in the ADR.

Our findings suggest mechanistic differences in the way the brain responds to increasing cerebral NO, depending on the severity of the injury. The no-DCI group responded as expected, demonstrating a move toward a less ischemic picture as demonstrated by increased ADR in response to NO repletion. However, the opposite was seen in the DCI group.

There are several potential explanations for these findings. One possibility is that there may be more severe brain injury and greater cerebral NO pathway dysfunction in patients who subsequently develop DCI. The dose of sodium nitrite chosen may have been insufficient for eliciting a response in the electroencephalography. A longer duration of infusion or a higher dose may have demonstrated different electroencephalographic changes.

A further possibility is that increased cerebral NO may selectively vasodilate in areas where there is less tissue damage, diverting blood away from the more ischemic areas, causing a “steal” phenomenon. This would cause a deterioration in perfusion to injured areas and a move toward a more ischemic pattern on electroencephalography. It also implies loss of local autoregulation, already known to occur after severe SAH and which is linked to poor outcomes (26). In addition, delivering NO to areas of the brain with increased levels of free radicals may encourage the production of neurotoxic peroxynitrite, further contributing to cell damage (27). It is important to note that the two groups of patients were indistinguishable at presentation in terms of clinical severity (WFNS score), Fisher grade, or baseline ADR. Therefore, increasing cerebral NO unmasks cerebral neuronal and metabolic dysfunction that is otherwise not detectable. Electroencephalographic changes have been previously linked to the development of DCI but have required several days of recording (15, 28).

Using a drug to probe electroencephalographic responses dynamically enabled the duration of recording to be considerably shorter than previous studies (average of 5 d; range 1–60 d) (29).

TCD and ICP recordings remained stable in response to sodium nitrite, confirming that the observed qEEG changes were not because of changes in global cerebral blood flow. The patients who developed DCI were not followed up with repeat TCD or other measurements outside routine clinical care to detect angiographic vasospasm, but this would be an interesting addition to any future studies. The small drop in MAP is very unlikely to have clinical implications.

### Limitations

The number of patients recruited was small, and there is risk of bias because of the unblinded nature of this study. A double-blinded and randomized validation study in a larger group of patients is necessary. As sodium nitrite was infused for only hour, it is possible that steady state might not have been achieved in all patients. Future studies might investigate longer recording durations and investigate the electroencephalographic changes during the offset of sodium nitrite. It would also be important to investigate patients with a less severe degree of EBI by including WFNS grades 1 and 2 in future work.

Although there was no control group, comparing baseline to values collected during the infusion allowed each patient to act as their own control, minimizing the effects of metabolic alterations, ICP changes, or effects of sedation. Although sedation will have affected the electroencephalographic pattern, it is unavoidable when studying this cohort of patients. The limited number of electroencephalographic electrodes hinders interpretation of spatial resolution, but fewer electroencephalographic electrodes increase the practicability of using this method in an intensive care setting.

## CONCLUSIONS

In conclusion, we have shown that a 1-hour infusion of IV sodium nitrite can induce measurable qEEG changes capable of discriminating which patients eventually develop DCI. Our findings emphasize the importance of EBI as a window of therapeutic opportunity to institute aggressive neuroprotective strategies. Measuring qEEG responses to an NO donor, such as sodium nitrite, might also represent a potentially useful method for patient stratification, which may be useful in clinical trials. Therefore, with further validation, these findings demonstrate the potential to develop an electroencephalography-based, patient-specific tool to predict DCI.

## ACKNOWLEDGMENTS

We thank Dr. Daniel Lunn for his invaluable advice regarding the statistical methods used in this article, Dr. David Garry for his comments on a previous version of this article, and Dr. Hilary Madder for supporting the study.

## Supplementary Material
