# Supplementary material for: Electroencephalographic Response to Sodium Nitrite May Predict Delayed Cerebral Ischemia After Severe Subarachnoid Hemorrhage
Source: Crit Care Med. 2016 Oct 14;44(11):e1067–73. doi: 10.1097/CCM.0000000000001950 (PMC5068187; doi:10.1097/CCM.0000000000001950)
Supplement: Supplementary file 1 [file ccm-44-e1067-s001.docx]

**EEG response to sodium nitrite may predict delayed cerebral ischaemia after severe subarachnoid haemorrhage - Supplementary information**

Payashi S Garry, FRCA ^1,2^; Matthew J Rowland, DPhil, FRCA^1,2^; Martyn Ezra, FRCA ^1,2^; Mari Herigstad, DPhil^1,3^; Anja Hayen DPhil^1,4^; Jamie W Sleigh, MD^5^; Jon Westbrook, FRCA^1,2^; Catherine E Warnaby, PhD ^1^; Kyle TS Pattinson, DPhil, FRCA^1,2^

1 = Nuffield Department of Clinical Neurosciences, University of Oxford, OX3 9DU UK

2 = Neurosciences Intensive Care Unit, Oxford University Hospitals NHS Trust, John Radcliffe Hospital, Oxford, OX3 9DU, UK

3 = Department of Clinical Health Care, Oxford Brookes University, Oxford, OX3 0BP UK

4 = School of Psychology & Clinical Language Sciences, University of Reading, RG6 6UR, UK

5 =Department of Anaesthesia, University of Auckland, Waikato Hospital, Hamilton 3240, New Zealand

Corresponding author and address for reprints:

Dr PS Garry

Nuffield Department of Clinical Neurosciences

John Radcliffe Hospital, Oxford, OX3 9DU

Tel: + 44 (0) 1865 572878

Fax: + 44 (0) 1865 234699

[payashi.garry@ndcn.ox.ac.uk](mailto:payashi.garry@seh.ox.ac.uk)

**Contents**

1. Explanation of statistical models used
2. Reported multilevel model
3. Bayesian model using baseline √ADR $(\sqrt{{ADR}_{0}})$as explanatory varible
4. Mathematics of back transformation
5. Figure I - Qq plot of model residuals
6. Figure II - Standardised vs fitted values for REML fit of random-effects model
7. Figure III – Showing fit of both multilevel and Bayesian model to the data
8. Table II – Levels of sedative drugs and vasopressor levels
9. Figure IV – Topographical representation of ADR changes
10. **Explanation of statistical methods used**

Our data comprised repeated measurements of the alpha/delta power ratio, five measures taken before drug infusion (‘baseline’ or 0) and five taken during the infusion (‘nitrite’ or 1) on fourteen patients. Seven of the patients later developed delayed cerebral ischaemia (DCI).

Age, severity of subarachnoid haemorrhage (SAH) as measured by the World Federation of Neurosurgeons (WFNS) grade and drug sedation levels are all potential confounds of these measures. Therefore, our model needed to account for possible effects of these variables on the ADR response to the drug.

In order to use the ADR as a response, it was necessary to obtain normality and homoscedasticity by transforming ADR by taking its square-root.

1. **Reported model – Multilevel modelling**

The model reported in the results fitted $\sqrt{ADR}$ as response, distinguished between the baseline measures and measures taken during the nitrite infusion with an indicator variable (0 or 1) and used patient ID (1-14) to identify repeated measures (making a multilevel model mandatory). Although it might be thought that this could have been avoided by fitting $\sqrt{{ADR}_{nitrite}}$ $- \sqrt{{ADR}_{baseline}}$ as the response in a single-level model, that would have led to an unjustified and incorrect assumption that the difference did not depend upon the baseline value. This is shown by the strong correlation between $\sqrt{{ADR}_{nitrite}}$ and $\sqrt{{ADR}_{baseline}}$ (ρ = 0.664).

The effects of subsequent development of DCI, propofol and midazolam on the ADR were taken into account. This showed no significant effect of propofol, midazolam, log(age) or WFNS grade in the fitted model, i.e. there was no evidence of an effect of these variables on the response variable.

The nitrite effect on the patients that subsequently developed DCI was significantly different from the drug effect on the non-DCI patients. The model had an R^2^ of 0.80, indicating that approximately 80% of the variability was accounted for.

*Output data from model*

|  | Value | Std.Error | DF | t-value | p-value |
| --- | --- | --- | --- | --- | --- |
|  |  |  |  |  |  |
| (Intercept) | 0.309 | 0.532 | 121 | 0.581 | 0.56 |
| DCI | 0.038 | 0.036 | 9 | 1.046 | 0.32 |
| Drug | 0.049 | 0.005 | 121 | 8.902 | <0.0001^**^ |
| Propofol | 0.0003 | 0.0002 | 121 | 1.584 | 0.12 |
| midazolam | 0.004 | 0.004 | 9 | 0.992 | 0.35 |
| log(Age) | -0.029 | 0.134 | 9 | -0.215 | 0.83 |
| WFNS.grade | -0.012 | 0.028 | 9 | -0.418 | 0.67 |
| DCI:Drug | -0.064 | 0.007 | 121 | -8.614 | <0.0001^**^ |

Intercept = the effect on the ADR response in the absence of drug or DCI (ie baseline in the patients who did not develop DCI)

DCI = the effect of development of DCI on the baseline ADR response (ie the effect of development of DCI in the absence of the drug)

Drug = the effect of sodium nitrite on the baseline ADR response

DCI:Drug = interaction term showing the effect of subsequent development of DCI on the ADR during the drug infusion

** = significant at 5% level

*Between group contrasts*

To obtained the between group contrasts, the model was ‘re-levelled’ to account for all combinations of DCI and drug. This involved adjusting the baseline (intercept) for the absence (0) or presence (1) of nitrite, and the absence (0) or presence (1) of subsequent DCI.

| Group | DCI 0: Baseline | DCI 0:  Nitrite | DCI 1:  Baseline | DCI 1:  Nitrite |
| --- | --- | --- | --- | --- |
|  |  |  |  |  |
| DCI 0:Baseline |  | <0.0001** | 0.072 | 0.186 |
| DCI 0:Nitrite | <0.0001** |  | 0.918 | 0.703 |
| DCI 1:Baseline | 0.072 | 0.918 |  | 0.006** |
| DCI 1:Nitrite | 0.186 | 0.703 | 0.006** |  |

DCI 0 = no DCI, DCI 1 = DCI,

Values are p values for each contrast. ** = significant at 5% level

This model takes into account the fact that baseline ADR is itself a random variable and therefore subject to measurement errors.

Quantile-quantile and plots of fitted vs standardised residuals demonstrating that this model fits the data very well are shown in Figures I and II below.

1. **Bayesian model fitting** $\sqrt{\boldsymbol{ADR}_{\boldsymbol{0}}}$**as an explanatory random variable**

As can be seen from the tables above, there is a trend towards a higher baseline ADR in the patients that developed DCI versus those that did not (*p =* 0.072). This raises the possibility that the baseline ADR was predictive of the ADR response to the drug, and that if the starting ADR was taken into account as an explanatory variable, this could possibly reduce or negate the drug effect seen and account for the observed results. Therefore, we remodelled the data to account for this.

In order to fit the baseline ADR as an explanatory random variable, it was necessary to use Bayesian model fitting. This is because the baseline ADR is a random variable in itself, with a highly significant effect on the ADR response variable as shown above. The linear regression multilevel modelling used above does not give an assessment of the magnitude of the effect whereas the Bayesian model does.

Therefore using the “JAGS” library in R, a full model with all covariates was fitted, with 30,000 MCMC iterations of which the first 10,000 were used as a burn-in and discarded in the summary statistics.

The model is:

$$\sqrt{{ADR}_{nitrite}}=b_{0}+ b_{1}\sqrt{{ADR}_{baseline}}{+ b}_{2}DCI+ b_{3}Propofol{+ b}_{4}midazolam{+ b}_{5}\log\left( Age \right){+ b}_{6}WFNS.grade+ b_{7}DCI:\sqrt{{ADR}_{baseline}}$$

estimate sd.err 2.5% 25% 50% 75% 97.5%

b0 0.043 0.179 -0.305 -0.077 0.043 0.162 0.396

b1 1.221 0.149 0.933 1.129 1.220 1.315 1.502

b2 0.029 0.055 -0.077 -0.009 0.028 0.065 0.137

b3 0.000 0.000 0.000 0.000 0.000 0.000 0.000

b4 0.001 0.001 -0.001 0.000 0.001 0.002 0.004

b5 -0.015 0.047 -0.109 -0.047 -0.016 0.016 0.077

b6 0.005 0.008 -0.011 -0.001 0.005 0.010 0.020

b7 -0.476 0.241 -0.959 -0.637 -0.476 -0.309 -0.017

sigma 0.029 0.005 0.019 0.026 0.030 0.033 0.040

sigma0 0.026 0.007 0.016 0.022 0.025 0.030 0.040

sigma is the estimated standard error of the underlying normal distribution of the response; sigma0 is the estimated standard error of $\sqrt{{ADR}_{baseline}}$.

Dropping the terms where the 95% credible interval contains zero we reduce the model to:

$$\sqrt{{ADR}_{nitrite}}=b_{0}+ b_{1}\sqrt{{ADR}_{baseline}}{+ b}_{2}DCI+ b_{7}DCI:\sqrt{{ADR}_{baseline}}$$

estimate sd.err 2.5% 25% 50% 75% 97.5%

b0 0.004 0.026 -0.042 -0.013 0.004 0.020 0.052

b1 1.273 0.137 1.026 1.190 1.279 1.362 1.514

b2 0.050 0.041 -0.029 0.023 0.050 0.078 0.132

b7 -0.558 0.184 -0.927 -0.680 -0.556 -0.435 -0.195

sigma 0.029 0.005 0.018 0.025 0.029 0.032 0.039

sigma0 0.026 0.007 0.016 0.022 0.025 0.029 0.039

Figure III confirms that the Bayesian and multilevel models are both fitting well and telling the same story - the multilevel plot uses the estimates from the reduced model given below.

Value Std.Error DF t-value p-value

(Intercept) 0.183 0.021 122 8.586 0.000

DCI 0.055 0.030 12 1.816 0.094

Drug 0.052 0.005 122 9.846 0.000

DCI:Drug -0.066 0.007 122 -8.938 0.000

The b1 coefficient shows that nitrite ADR depends significantly on baseline ADR, which is to be expected. However, the DCI:$\sqrt{{ADR}_{baseline}}$ interaction term gives the correction to the dependence on baseline-ADR and has the effect of modifying the coefficient from 1.273 down to 1.273 − 0.558 = 0.715. This is a significant (*p* < 0.0001) reduction to 56% of its previous value and shows the effect of DCI on the nitrite ADR score.

In terms of nitrite-baseline difference, for no DCI:

$$\sqrt{{ADR}_{nitrite}}- \sqrt{{ADR}_{baseline}}=0.004+0.273\sqrt{{ADR}_{baseline}}$$

whilst for the patients that subsequently developed DCI:

$$\sqrt{{ADR}_{nitrite}}- \sqrt{{ADR}_{baseline}}=0.004+0.050+\left( 0.273-0.558 \right)\sqrt{{ADR}_{baseline}}= 0.054-0.285\sqrt{{ADR}_{baseline}}$$

Therefore this model shows that in the no DCI group, the baseline ADR increases by 0.273$\sqrt{{ADR}_{baseline}}$ in response to sodium nitrite, whereas in the DCI group the baseline ADR decreases by $0.054-0.285\sqrt{{ADR}_{baseline}}$ in response to sodium nitrite, confirming the results of the multilevel model.

1. **Mathematics of the back transformation**

In order to satisfy the normal and homoscedasticity requirements for valid fitting of a linear model, a transformation of the original response variable *y* to a suitable response variable, say *z*, had to be carried out. Suppose further that, to return to the original variable, the inverse transformation is $y=f\left( z \right).$ By Taylor expansion about the mean $\mu_{z}$ it can be shown that, to order $n^{-1}$ where $n$ is the sample size,

$$\mu_{y}\simeq f\left( \mu_{z} \right), \sigma_{y}\simeq\sigma_{z}f'\left( \mu_{z} \right),$$

where $f'$ is the first derivative of $f$.

We therefore applied a back transformation to the results obtained above to enable calculation of the absolute ADR values and standard errors resulting from this model.

1. **Figure I**

Quantile-quantile plot of the calculated quantiles from the linear model vs the sample quantiles from the data. This is a probability plot which demonstrates that the probablility distribution of the fitted linear model is very similar to the probablility distribution of the data, confirming that the calculated model is a good fit for the data.

1. **Figure II**

Demonstration that variation in residuals for the linear model reported are unbiased and homoscedastic. This provides further confirmation that the model is a good fit for the data.

1. **Figure III**

Demonstration of the fit of both models to the data. X axis represents values from the data. Y axis demonstrates the values as predicted by the model. It can be seen that both models show a strong linear correlation (red line) between actual and predicted values.

1. **Table II**: Sedation and vasopressor levels for each patient, both pre and during sodium nitrite infusion.

| Patient | Propofol  (mg/hr) | | Fentanyl (mcg/kg/hr) | | Midazolam  (mg/hr) | | Atracurium  (mg/hr) | | Noradrenaline (mcg/kg/min) | |
| --- | --- | --- | --- | --- | --- | --- | --- | --- | --- | --- |
|  | Pre | During | Pre | During | Pre | During | Pre | During | Pre | During |
| 1 | **0** | **0** | **200** | **200** | **10** | **10** | **0** | **0** | **0.34** | **0.34** |
| 2 | **0** | **0** | **300** | **300** | **20** | **20** | **50** | **50** | **0.38** | **0.38** |
| 3 | **200** | **200** | **150** | **150** | **0** | **0** | **0** | **0** | **0** | **0** |
| 4 | **0** | **0** | **250** | **250** | **10** | **10** | **0** | **0** | **0.16** | **0.16** |
| 5 | **180** | **200** | **0** | **0** | **0** | **0** | **0** | **0** | **0.17** | **0.20** |
| 6 | **100** | **100** | **150** | **150** | **0** | **0** | **0** | **0** | **0.15** | **0.15** |
| 7 | **100** | **100** | **200** | **200** | **0** | **0** | **0** | **0** | **0** | **0** |
| 8 | **0** | **0** | **100** | **100** | **0** | **0** | **0** | **0** | **0.14** | **0.14** |
| 9 | **220** | **230** | **200** | **250** | **0** | **0** | **0** | **0** | **0.25** | **0.25** |
| 10 | **0** | **0** | **150** | **150** | **0** | **0** | **0** | **0** | **0.03** | **0.03** |
| 11 | **150** | **150** | **100** | **100** | **0** | **0** | **0** | **0** | **0.15** | **0.16** |
| 12 | **180** | **180** | **200** | **200** | **0** | **0** | **0** | **0** | **0.20** | **0.20** |
| 13 | **150** | **200** | **200** | **200** | **0** | **0** | **0** | **0** | **0** | **0** |
| 14 | **200** | **200** | **150** | **150** | **0** | **0** | **0** | **0** | **0.13** | **0.13** |

mg/hr = milligrams per hour; mcg/kg/hr = micrograms per kilogram per hour; mcg/kg/min = micrograms per kilogram per minute

1. **Figure II**

Topographical representation of power changes before and during infusion of sodium nitrite in each patient. Location of aneurysm is shown beside each patient. Due to the number of electrodes that it was practicable to use it is difficult to find a consistent pattern of EEG changes related to the aneurysm location. R = right; L= left; ACom = anterior communicating, PCom = posterior communicating; MCA = middle cerebral artery
